# When Blood Is Thicker Than Water: A Case of Acute Pancreatitis Secondary to Familial Hypertriglyceridemia

**DOI:** 10.7759/cureus.51511

**Published:** 2024-01-02

**Authors:** Krishna Patel, Nitya Devireddy, Calista Long, Annika Daya, John Cherneskie, Kaleigh Krill

**Affiliations:** 1 Department of Medicine, Penn State College of Medicine, Hershey, USA; 2 Department of Medicine, Penn State Health Milton S. Hershey Medical Center, Hershey, USA; 3 Department of Medicine, Veterans Affairs Medical Center, Lebanon, USA

**Keywords:** hypertriglyceridemia, hypertriglyceridemia-induced pancreatitis, familial hypercholesterolemia, hypertriglyceridemic pancreatitis, familial lipid disorders, acute pancreatitis

## Abstract

Hypertriglyceridemia is one of the major causes of acute pancreatitis in addition to gallstones and alcohol use. These etiologies are often associated with underlying comorbidities. Acute pancreatitis secondary to hypertriglyceridemia is associated with an increase in clinical severity and further complications. We present a case of a 56-year-old man with a past medical history of hypertension, diabetes mellitus, and familial hypertriglyceridemia who was diagnosed with acute pancreatitis secondary to hypertriglyceridemia. The patient presented with 9/10 pressure across the abdomen radiating to the sternum. Labs revealed elevated triglyceride count > 8000 mg/dL and cholesterol > 705 mg/dL. Abdominal CT showed fat stranding along the anterior aspect of the pancreatic head. The patient was managed with IV fluids, nil per os (NPO), and statin management for hypertriglyceridemia. Seven days later, triglycerides decreased to 658 mg/dL, and abdominal pain resolved. This case highlights an unusual presentation of acute pancreatitis and demonstrates the importance of understanding the spectrum of etiologies for this condition.

## Introduction

Hypertriglyceridemia is one of the major causes of acute pancreatitis, accounting for up to 10% of all cases [1]. It typically occurs in patients with dyslipidemia in the presence of a secondary condition, such as inadequately controlled diabetes, excess alcohol consumption, or medication use. It is also associated with greater clinical severity and rate of complications compared to other etiologies of acute pancreatitis [1]. Familial hypertriglyceridemia is characterized by an increase in very low-density lipoprotein (VLDL) particles and follows an autosomal dominant inheritance pattern [2]. Here, we discuss a patient case of acute pancreatitis secondary to familial hypertriglyceridemia.

This article was previously presented as a meeting abstract and poster at the 2023 PA-ACP Eastern Region Conference on October 21, 2023.

## Case presentation

A 56-year-old male with hypertension, uncontrolled diabetes mellitus (A1C: 12.6%), and familial hypertriglyceridemia presented to the emergency department with a chief concern of abdominal pain for two days. The pain was described as 9/10 pressure radiating across the abdomen to the sternum. The patient stopped taking his prescribed medications one year prior, including amlodipine 10 mg daily, atorvastatin 20 mg daily, fish oil 1000 mg twice per day (BID), cholecalciferol 50 mcg daily, vitamin B12 500 mcg daily, lisinopril 40 mg daily, and metformin 1000 mg BID.

**Figure 1:**
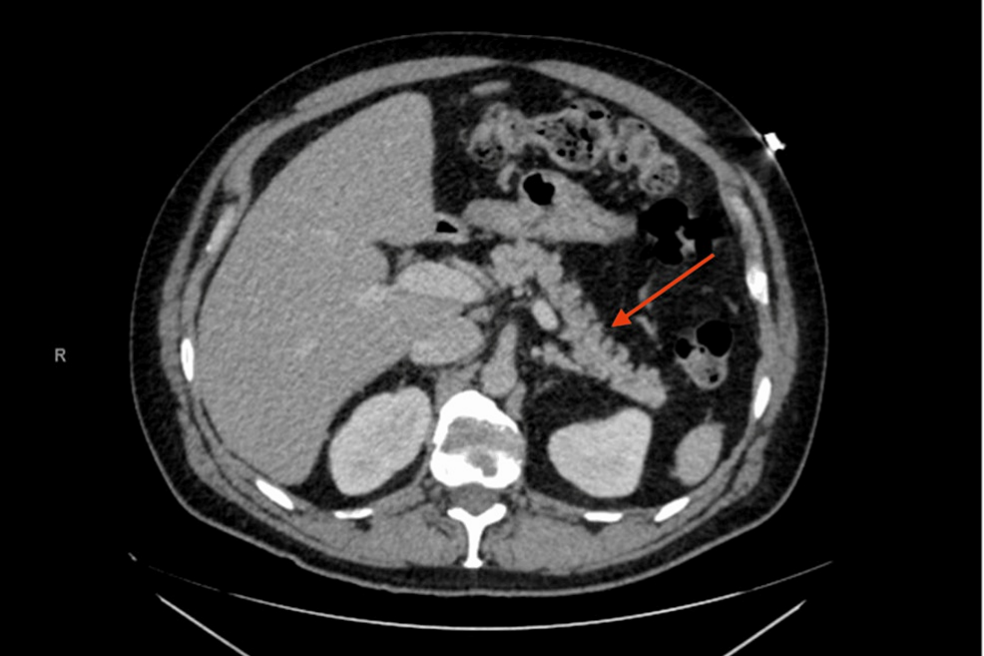
Axial computed tomography scan showing fat stranding along the pancreas head.

Physical exam was unremarkable aside from tenderness to palpation diffusely throughout the abdomen. Blood drawn for labs had a white hue and was noted to be “strongly lipemic.” Labs revealed elevated triglyceride count > 8000 mg/dL, cholesterol > 705 mg/dL, and lactic acid was 5.0 mmol/L. Glucose was elevated to 403 mg/dL, sodium decreased to 114 mEq/L, and lipase elevated to 298 U/L. Liver function tests (LFTs) were within normal limits. Abdominal CT showed fat stranding along the anterior aspect of the pancreatic head (Figure 1), confirming the diagnosis of acute pancreatitis secondary to familial hypertriglyceridemia based on the Atlanta classification [3]. The revised Atlanta classification requires that two or more of the following must be met to diagnose acute pancreatitis: (1) abdominal pain (i.e., right upper quadrant pain) suggestive of pancreatitis; (2) serum amylase or lipase levels on labs greater than three times the upper limit of normal; and/or (3) characteristic imaging findings on CT, often described as pancreatic inflammation or fat-stranding on the pancreas.

The patient was managed with insulin drip until triglycerides were <500 mg/dL, IV fluids to slowly correct sodium, bowel rest with nil per os (NPO) initially, restarting low-fat diet as tolerated, and restarted on anti-lipid management for hypertriglyceridemia. Medications at discharge included atorvastatin 40 mg daily, fenofibrate 145 mg daily, fish oil 2000 mg BID for medium-chain fatty acids, lisinopril 10 mg daily, basal insulin, and metformin 500 mg BID.

## Discussion

Acute pancreatitis is a disease that has many different etiologies, the most common of which include alcohol and gallstones, followed by hypertriglyceridemia-mediated disease. In gallstone pancreatitis, the most common cause of acute pancreatitis in the Western world, blockage of the pancreatic duct by a biliary stone leads to inflammation of the pancreas [4]. Alcohol-induced pancreatitis is less understood as ethanol itself does not directly cause pancreatitis. Rather, it sensitizes the pancreas to injury by mechanisms such as a high lipid diet, cigarette smoke, and infectious agents [5]. While acute disease is common, oftentimes this can progress to chronic pancreatitis.

The proposed mechanism for hypertriglyceridemia-induced pancreatitis suggests that high levels of lipids increase plasma viscosity, resulting in ischemia and inflammation of pancreatic tissue [1]. Pancreatitis secondary to hypertriglyceridemia typically occurs in those with genetic lipid disorders, most commonly types I, IV, or V, as these result in higher levels of lipids and triglyceride-rich chylomicrons (chylomicronemia) [1]. In type I, chylomicron metabolism is predominantly affected, resulting in chylomicronemia [1]. Patients with type IV, familial combined hyperlipidemia, present with elevated VLDL levels, whereas those with type V are characterized by elevated VLDL and chylomicrons due to gene alterations that reduce catabolism [1]. Type IV and V are both more prevalent than type I and are more affected by environmental risk factors, such as obesity, alcoholism, and diet [1].

In the setting of hypertriglyceridemia, pseudohyponatremia should be suspected as the increased mass of the non-aqueous lipid components of serum can dilute the aqueous component of serum, subsequently reducing plasma sodium concentration [6]. A proper workup is necessary to determine the etiology of pancreatitis. Liver function tests, lipid panels, and pancreatic enzymes are important laboratory values to obtain. Right upper quadrant ultrasound is an important step to identify obstructive causes if indicated. Magnetic resonance cholangiopancreatography (MRCP) and abdominal CT may also aid in workup. All patients should be managed with aggressive IV fluids, early enteral feeding to advance as tolerated, and pain medication, with further management to control the underlying etiology.

Management of familial hypertriglyceridemia will help in preventing the recurrence of this disease. These treatments are primarily focused on reducing levels of triglycerides, managing comorbid conditions, and lifestyle modifications such as diet and exercise [7]. Statins are often the first-line pharmacotherapy choice. Fibrates such as fenofibrate and gemfibrozil may also be used for the management of hypertriglyceridemia [8]. Fibrates have been shown to reduce triglyceride levels by up to 50%, attributing their placement in the typical treatment protocol [8]. Other medications such as niacin and fish oil (omega-3 fatty acids) have been shown to reduce both VLDL and triglyceride levels [9].

## Conclusions

Acute pancreatitis secondary to hypertriglyceridemia makes up a sizable proportion of cases of pancreatitis within the country. In this case report, we describe a case of acute pancreatitis secondary to familial hypertriglyceridemia in a 56-year-old man. The patient presented with abdominal pain, found to have triglyceride count > 8000 mg/dL and cholesterol > 705 mg/dL, with abdominal CT showing fat stranding along the anterior aspect of the pancreatic head. The patient was successfully treated using IV fluids, NPO, and statin management for hypertriglyceridemia, resulting in a steady decline of his triglycerides to 658 mg/dL and resolution of abdominal pain. Prevention of further complications depends on the management of the underlying condition of familial hypertriglyceridemia. It is because of this that it is important to have early identification and the need for high clinical suspicion for acute pancreatitis to prevent further complications. This case demonstrates an unusual presentation of acute pancreatitis caused by familial hypertriglyceridemia, highlighting the importance of recognition of broad presentation patterns and etiologies of acute pancreatitis.
